# Effectiveness of Interventions to Prevent Musculoskeletal Disorders among District Hospital Nurses in Vietnam

**DOI:** 10.1155/2022/1539063

**Published:** 2022-03-10

**Authors:** Thi Tham Nguyen, Thanh Hai Nguyen, Duc Luan Hoang, Thi Giang Hoang, Minh Khue Pham

**Affiliations:** ^1^Faculty of Public Health, Haiphong University of Medicine and Pharmacy, Vietnam; ^2^Phu Tho College of Medicine and Pharmacy, Vietnam

## Abstract

**Background:**

Nurses are one of the population groups with the highest prevalence of musculoskeletal disorders (MSDs). Preventive measures in Vietnamese hospitals on the job have not been proposed to study their effectiveness due to barriers related to the lack of knowledge about MSDs by health care administrators and the lack of human resources with expertise in MSD management in hospitals.

**Objectives:**

This study is aimed at evaluating the effectiveness of basic interventions (education, physical exercise) to prevent MSDs among district hospital nurses in Vietnam. *Material and Methods*. A quasi-experimental study was carried out before/after over a period of one year among two groups of nurses, one receiving the intervention (*n* = 162) and the other the control group (*n* = 128). The intervention includes 3 components: training on MSDs, ergonomics training, and instructions for physical exercise. The pre- and postintervention assessment tools included the Modified Nordic, Quality of Life Enjoyment and Satisfaction Short-Form (Q-LES-Q-SF), and the Kessler Psychological Distress Questionnaire (K6). A generalized estimating equation analysis was performed to assess the difference between the two groups at two points in time (before and after the intervention) on some indicators (prevalence of MSDs in the last 12 months and 7 days, score for quality of life and psychological distress).

**Results:**

There was a significant difference of the test on the prevalence of MSDs in the last 7 days between the 2 groups before and after the intervention with the *p* value = 0.016. This difference occurred in 4 anatomical sites: neck, shoulder/upper arm, wrists/hand, and lower back, with *p* values being 0.013, 0.011, 0.038, and 0.009, respectively.

**Conclusions:**

The intervention measures are probably effective in reducing the prevalence of MSDs at 4 anatomical sites in the last 7 days. More in-depth studies are needed with a combination of measures over a longer period of time to obtain stronger evidence of interventions.

## 1. Introduction

The global nursing workforce is 27.9 million and is the largest occupational group in the health sector, accounting for approximately 59% of health professions [1]. In this profession, nurses encounter a variety of occupational health problems such as biological hazards (hepatitis B, hepatitis non-A non-B, tuberculosis, AIDS…), chemical hazards (cytotoxic drugs, anesthetic agent, antibiotics, formaldehyde, ethylene oxide…), psychosocial hazards (stress, shift work, suicide…), and physical hazards (needle stick injury, back pain and back injuries, radiation…) [2], especially musculoskeletal disorders (MSDs) with the prevalence of 71.9% [3]. Many studies have shown that the nursing profession is influenced by many environmental, working conditions, mental factors, and even personal factors that can contribute to MSDs [4, 5]. A review of the literature by Soylar and Ozer demonstrated that cumulative trauma and repetitive tasks included lifting, transferring, or repositioning patients; prolonged standing and also awkward postures (bending, lengthen) were highly associated with MSDs in nurses. These work-related health problems were also significantly associated with age, sex, BMI, type of service worked, shift work, and hospital work. Studies have also shown that MSDs are mostly seen in operating room and intensive care nurses [6].

In developed countries around the world, there are many studies on MSD among nurses. On the contrary, in developing countries, including Vietnam, there are few published data. Several recent studies in Vietnam showed the high prevalence of MSDs in general [7] and of multiple musculoskeletal symptoms (MMS) [8] with the abundance of risk factors in hospital nurses. Another study has shown a negative impact of MSDs at multiple sites on the quality of life of nurses [9].

From that, it is clear that the burden of MSDs on nurses in Vietnam is large. This burden will affect their health and quality of life, thereby affecting the quality of their patient care. Therefore, it is necessary to take preventive measures systematically and comprehensively to reduce this burden. However, in Vietnam at present, the prevention of MSDs in nurses still faces many difficulties and barriers in implementation. There are many causes for this situation. Firstly, the field of occupational health in Vietnam in general has not received much attention and research. Second, occupational-related MSDs have not been included in the list of covered occupational diseases in Vietnam [10]. Third, there is a lack of suitable scientific evidence from studies on the feasibility and effectiveness of MSD interventions in nurses. For these reasons, the feasibility of intervention implementation is limited due to not being able to convince the experts, organizers, or even the trust of nurses about the effectiveness of interventions. A recent study by Khue et al. has shown that the lack of knowledge on MSDs by health care administrators inside and outside the hospitals and the lack of human resources with expertise in MSD management are important barriers to the implementation of an MSD prevention program in Vietnamese hospitals [11].

To address these issues, it is necessary first of all to obtain appropriate scientific evidence from studies on the effectiveness of preventive measures against MSDs in Vietnamese nurses.

In the context that the field of occupational health in Vietnam is still underdeveloped, and resources (human and material) for activities in this field are limited, the question arises as can simple and highly feasible interventions improve MSDs in nurses? If yes, how effective are those measures? In other words, what is the effect of basic interventions on MSDs on nurses? From there, it is the basis for further research in the future or for suggestions and recommendations for leaders in hospitals about the importance of preventing MSDs for their staff. This is the main reason why this study was conducted.

Haiphong is one of the largest cities in Vietnam with a very high population density (1.274/km^2^), but human resources for the health sector are short of supply with 7.7 doctors and 17.6 nurses per 10.000 inhabitants [12]. Therefore, the work pressure of medical personnel, especially nurses, is very high to meet the high demand for medical care of people. The evaluation of health risks to medical personnel and the effectiveness of preventive measures will contribute to improving their health status, thus improving the quality of health care services.

This study will answer whether the basic interventions (education, physical exercise) to prevent MSDs among district hospital nurses in Haiphong, Vietnam, are feasible, effective, or not and how effective are they?

## 2. Materials and Methods

A quasi-experimental before/after study was carried out over a period of one year between two groups of district hospital nurses, one receiving the intervention (intervention group) and the other being the control group.

The total number of district public hospitals in Haiphong is 15, divided into 2 types: 8 rural district hospitals (located in rural areas) and 7 urban district hospitals (located in urban areas).

Calculation of the number of subjects is needed.

The minimum sample size per group for a before/after intervention study was calculated using the formula [13].

(1) $$\begin{array}{c} n=2{\left(Z_{\alpha /2}+Z_{\beta }\right)}^{2}\frac{p\left(1-p\right)}{{\left(p_{1}-p_{2}\right)}^{2}}, \end{array}$$

where*α*is 0.05 ➔*Z*_*α*/2_is 1.96;*β*is 0.10 ➔*Z*_*β*_is 1.28;*p*_1_is the expected prevalence of MSDs in the controlgroup = 81%or 0.81 (depending on the results of a previous study) [11];*p*_2_is the expected prevalence of MSDs in the case group after theintervention = 60%or 0.60;*p*is the pooled prevalence=(*p*_1_ + *p*_2_)/2 = (0.81 + 0.60)/2 = 0.705.

The minimum sample size for each group was 99 nurses.

### 2.1. Sampling Technique

**Figure 1:**
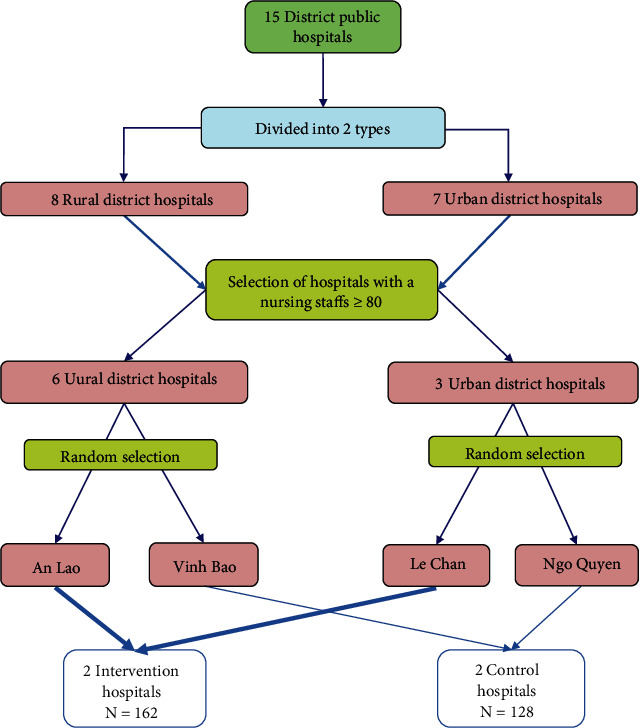
Flow chart of hospital selection.

First, a list of nurses the number of available (who have a nursing degree, who have worked in the hospital for at least 12 months immediately prior to the start of the study) was established for each hospital. The number of nurses in each hospital was divided into 2 thresholds, either less than 45 or greater than 80 (see Table 1). In addition, to obtain an appropriate sample regarding the hospital type, four hospitals were selected (two hospitals per group, and each group contains one urban district hospital and one rural district hospital). Furthermore, to the guarantee the minimum sample size per group (99 nurses), only hospitals having 80 nurses or more were selected. Therefore, there are nine hospitals that meet this condition, including six rural district hospitals (An Duong, An Lao, Kien Thuy, Tien Lang, Thuy Nguyen, and Vinh Bao) and three urban district hospitals (Hong Bang, Le Chan, and Ngo Quyen). These 9 hospitals were divided into 2 branches (depending on the type of hospital). Then, a hospital per branch was randomly selected (random draw) for each group. The two hospitals, Le Chan (urban district) and An Lao (rural district), were finally chosen for the intervention group, while the control group included hospitals in Ngo Quyen (urban district) and Vinh Bao (rural district) (see Figure 1).

The total number of nurses in the two hospitals that received the intervention was 210, of which 197/210 agreed to participate. However, at the time of the pre- and postintervention evaluation, only 162/197 nurses had fully participated in the evaluation. The reasons why the 35 nurses did not participate are maternity leave (18/35), training (14/35), and change of job or work position (3/35).

For the control group, the total number of nurses in the 2 hospitals was 260 people, of which 138 agreed to participate. During the survey, 128/138 fully participated from start to finish. The reasons given were similar to those of the intervention group: maternity leave [6], training [3], and change of job or position [1].

### 2.2. Research Instruments

The pre- and postintervention evaluation questionnaires included as follows:

1. *A Sociodemographic Questionnaire*. Collecting some personal information, such as gender, age, height, weight, seniority, and personal history of musculoskeletal diseases throughout their life
2. *The Standardized Nordic Questionnaire*. This questionnaire, which was developed by Kuorinka et al. in 1987 [14], evaluates the trouble (ache, pain, discomfort) of the locomotive organs at nine different positions on the body (neck, shoulder/upper arm, elbow/forearm, wrist/hand, upper back, lower back, hip/thigh, knee/lower leg, and ankle/foot) during the last 12 months and during the last 7 days and the impact of those problems on the work and life of the respondent
3. *The Short Form of the Quality of Life Enjoyment and Satisfaction Questionnaire (Q-LES-Q-SF)*. This was developed by Stevanovic et al. based on the original long-form Quality of Life Enjoyment and Satisfaction Questionnaire (Q-LES-Q) developed by Endicott et al. in 1993 [15]. The short form, with 14 elements, evaluates general enjoyment and satisfaction with physical health, mood, work, household activities, leisure activities, social and family relationships, daily functioning, sexual life, economic status, and general well-being. The questions were rated on a five-point scale (from “very poor” to “very good”). The scoring of the Q-LES-Q-SF involves all of the 14 items to yield a total score (range from 14 to 70) with higher scores indicating better quality of life and vice versa
4. *The Kessler Psychological Distress Scale (K6)*. This short questionnaire consists of six questions about a person's emotional state (nervous, hopeless, restless, or fidgety, so depressed that nothing can cheer you up, everything is an effort, and worthless) [16]. Each question is scored from 0 to 4 (from “None of the time” to “All of the time”). The total score is calculated by calculating the score from the six questions, with total ranging from 0 to 24. A higher score indicates a more serious level of psychological distress

These questionnaires were used by our researchers for direct interviews ranging from 30 to 45 minutes in length before and after intervention.

For the intervention period, the following tools related to MSDs and preventive measures were used:

- Presentations on MSDs, ergonomics, and physical exercise
- Documents and training material
- Leaflets, posters, and illustrations
- Instructional videos

### 2.3. Intervention Content and Implementation

The intervention contents were compiled based on the recommendations of the World Health Organization (WHO) on the prevention of MSDs on the workplace [17] and on the prevention of MSDs on the hospital sector of the General Directorate of Labor Humanization in Belgium [18] and the Guideline for ergonomics for the prevention of MSDs of the Occupational Safety and Health Administration, US Department of Labor [19]. The intervention includes 3 components:

- *Training on MSDs*. Providing knowledge on MSDs such as definitions, symptoms, consequences, and preventive measures. This was a presentation with MSDs that lasted about 30 to 45 minutes
- *Ergonomics Training*. Providing knowledge of ergonomics, showing nurses how to correctly perform professional operations in patient care such as wound care, patient lifting, support, and transfer, as well as when handling medical equipment such as stretchers, wheelchairs, beds, trolleys… This component includes two forms: a presentation on ergonomics (30 minutes) and a practical session on handling and correct postures (30 to 45 minutes)
- *Instructions for Physical Exercises*. Stretching exercises (or relaxation), musculation training exercises, and back mobilization exercises. This is a practical session lasting about 30 minutes

Nurses from the 2 intervention hospitals were trained for the first time during the first week of the first month of the intervention period. This training included 2 sessions:

- The first session, lasting about 60 to 75 minutes, contains two presentations (one on MSDs and the other on ergonomics)
- The second session also lasts from 60 minutes to 75 minutes, including 2 practice contents on ergonomics and physical exercise

These contents were repeated in the first weeks of the third and sixth month of the intervention period. Therefore, the intervention group received a total of three times of training. The last six months were the observation period. The aim was to see if the nurses were carrying out the training content correctly and fully. Leaflets, posters, and illustrations were introduced, distributed, and displayed in departments/services throughout the one-year intervention period.

### 2.4. Statistical Analysis

The SPSS version 22.0 software was used for data analysis. A chi-square test for qualitative variables and a Student's *t*-test for continuous variables were used to compare some characteristics between the intervention and control groups. A generalized estimating equation (GEE) analysis was performed to assess the difference between the two groups (intervention and control) at two points in time (before and after the intervention) on some indicators (prevalence of MSDs during 12 months, over the last 7 days, the score for quality of life and psychological distress), in the interaction of two variables (before/after intervention and intervention Yes/No), and adjusted for age, sex, BMI, and history of musculoskeletal diseases to control the impact of these variables on the model. The level of significance was set at a *p* value of less than 0.05.

## 3. Results

### 3.1. Profiles of Participants at Baseline

In this study, we carried out an intervention (full participation from start to finish) for 162 nurses and had a total of 128 nurses in the control group.  Table 2 shows that some general characteristics of age, sex, BMI, and history of musculoskeletal disease between the intervention group and the control group at baseline were quite similar. This has been shown by the *p* values in the Student's *t*-test and the Chi2 test, both higher than 0.05 (see Table 2).

### 3.2. Effectiveness of Interventions in the Prevalence of MSDs

To assess the effectiveness of the intervention, we used generalized estimating equation analysis. The purpose of this analysis was to assess the difference between the two groups (intervention and control) at two time points (before and after intervention) on some study indicators in the interaction of two variables (intervention before/after and intervention yes/no) and adjusted for age, sex, body mass index, and history of musculoskeletal diseases to control for the impact of these variables on the model.

Regarding impact on the prevalence of MSDs, there was a significant difference of the GEE's test on the prevalence of MSDs in the last 7 days between the 2 groups before and after the intervention with the *p* value = 0.016. In more detail, the prevalence of MSDs in the last 7 days in the control group was 1.9 times higher than in the intervention group after the intervention. For the prevalence of MSDs in the last 12 months, the test did not provide significance by showing that the *p* value is equal to 0.059 (see Table 3).

In Table 3, the results of the test found positive changes in the prevalence of MSDs in the last 7 days. We would therefore like to know where these changes occur among the 9 anatomical sites studied. Using the same analysis, we found significant changes in the test between the 2 groups at the 4 anatomical sites: neck, shoulder/upper arm, wrists/hand, and lower back with *p* values lower than 0.05 (0.013, 0.011, 0.038, and 0.009, respectively) (see Table 4). This means that the intervention measures are probably effective in reducing the prevalence of MSDs at these 4 anatomical sites.

For the remaining anatomical sites, this analysis showed that the *p* values were greater than 0.05.

### 3.3. Effectiveness of Interventions in Quality of Life and Psychological Distress Scores

It is the same explanation for the quality of life and the psychological distress; Table 5 has shown that there is no significant change of the GEE's test on the score of quality of life and on the score of psychological distress between the 2 groups before and after the intervention with *p* values greater than 0.05 (0.344 and 0.789, respectively) (see Table 5).

## 4. Discussion

This is a quasi-experimental before/after study to assess the effectiveness of interventions by measuring the prevalence of MSDs, quality of life, and psychological distress scores before and after the implementation of the intervention for the 2 groups (intervention and control) to compare each other. According to the methodological guide concerning quantitative methods for evaluating interventions aimed at improving the practices of the French High Authority of Health (Haute Autorité de Santé Française (HAS)), this design has certain limitations. The results of a before/after study may overestimate the effects of interventions due to preexisting trends in improvement or variations related to another cause than intervention [20]. Therefore, to limit these drawbacks, on the one hand, to create a contemporary controlled site, the hospitals were divided into two groups representing two different types of hospitals (rural and urban districts). On the other hand, a random selection step (randomized) was applied to select 2 intervention hospitals and 2 control hospitals among the 2 hospital groups above (described in detail in the sampling technique section). This work made the characteristics between the two hospital groups participating in the study relatively similar and comparable. In addition, thanks to the choice of group (hospitals), the risk of contamination (occurs between nurses if the intervention and the control group occur in the same hospital—the learning effect of colleagues) did not exist. However, this trial exposes to the risk known as the Hawthorne effect [21]: the nurses in the group having benefited from the intervention may have improved their behavior because they still think that they are part of the intervention group, and the reverse occurs with the control group. In addition to trying to limit the drawbacks of the before/after study, a generalized estimating equation was performed to limit confounding factors and make a direct change comparison between the intervention and the control group.

Before the study, the sample size for each group was calculated to ensure methodological optimization. The actual number of nurses who participated in the study ensured the minimum sample size condition. Then, four hospitals were selected so that each group had a different type of hospital (rural district and urban district) to see the representativeness of the sample. Furthermore, sociodemographic characteristics such as age, sex, BMI, and history of musculoskeletal disease were similar and comparable between the intervention and control groups (no statistically significant differences). These conditions guarantee a good sample for this study.

After one year of intervention, the prevalence of MSDs in the last 12 months, the quality of life, and the psychological distress score were not statistically different between the 2 groups before and after the intervention, although that most of the changes are positive. It is possible that in the control group, although the nurses did not receive any interventions, their knowledge after answering many questions about MSDs was also improved. For that reason, they can apply good practices and postures or apply the acquired knowledge themselves to limit the risk of MSDs. This explains why the prevalence improved after the intervention but was not statistically significant in the tests.

The GEE has pointed out significant positive changes that have only occurred in the prevalence of MSDs in the last 7 days for 4 anatomical sites (neck, shoulder/upper arm, wrist/hand, and lower back) between the 2 groups after intervention compared with before intervention. Work-related MSDs clearly are a chronic disease that develops and evolves over a long period of exposure to risk factors. Therefore, the process of developing preventive strategies up to the time of intervention with preventive measures takes time to improve or reduce musculoskeletal symptoms. Additionally, this study only applied certain simple preventive measures (education, training, and physical exercise), and the duration of the intervention was not long enough (6 months of intervention then 6 months of observation). All of these reasons are likely to cause positive changes, but not statistically significant, in the prevalence of MSDs in the past 12 months, in the obstructive work, in the quality of life score, and in the psychological distress score. However, it is undeniable that the interventions used were also significant effective in relieving musculoskeletal symptoms of certain anatomical sites more commonly in nurses (lower back, neck, shoulder, and wrist) but only in the last 7 days. Therefore, it is necessary to have more effective preventive measures and a longer intervention time in future studies.

Therefore, although there have been many studies and trial studies of various interventions aimed at preventing MSDs, current data still show a high prevalence of MSDs among nurses, even in countries with developed occupational disease prevention systems [22–24]. This raises questions about the effectiveness of these interventions and how to apply them effectively in the hospital setting for nurses. Most studies around the world, especially in developed countries, have shown greater effectiveness when combining multiple interventions in parallel [25, 26]. However, the quality of most of these studies was poor, and the quality of randomized controlled trials was very low [26–28]. These studies mentioned many different interventions, which can be divided into different groups:

1. Education and training on patient care [29–32]
2. Provision of assistive devices for patient support and care (patient lifting systems, provision of manual handling equipment, etc.) [33–35]
3. Individual measures (physical exercise, stretching exercise [36, 37], cognitive-behavioral therapy [38], wearing unstable shoes [39], or stress management [40])
4. Multicomponent intervention that includes two or more of the above interventions [25, 41, 42]

Regarding the application and feasibility of these measures to the practice of nurses, many elements must be taken into account to achieve the greatest efficiency, in particular for countries which are still limited in the prevention of occupational diseases. Ziam et al. examined the application of MSD prevention practices among nurses in Canada and identified organizational factors and sociodemographic variables that may or may not support [43]. In this survey, nurses stated that several factors that promote the application of preventive practices for MSDs in their work environment, including the availability of equipment in good condition for the transfer of patients (86%), training in MSD prevention practices (85%), and support for caregivers (85%), hold information sessions on the use of patient transfer and movement equipment (81%). In addition, several barriers to the implementation of preventive measures for MSDs are also reported: the lack of time to apply preventive measures, the lack of training, the unavailability of human resources (preventers, for example), the unavailability of efficient and sufficient patient transfer and movement equipment or the difficulty of accessing this equipment when needed, the discrepancy between training, and the reality of the work, as well as the lack of support of colleagues [43]. These results show that despite being a very developed country in terms of preventive measures in general and in the field of occupational health in particular, there are still limitations and difficulties in applying preventive measures for MSDs in the hospital setting. These difficulties also apply to Vietnam. The recommendations in the field of occupational health, as well as strategies for the prevention of occupational diseases for workers in general and for health workers in particular, there are still many limitations in Vietnam. In particular, the application of preventive measures in hospitals remains difficult when, on the one hand, the daily workload of nurses is essential with high work pressure [44], and on the other hand, there are barriers to the establishment of an MSD prevention program in Vietnamese hospitals due to poor recognition of the importance of the MSD problem by hospital managers, as well as lack of human resources with expertise in the field of MSD prevention [11]. For this reason, in this pilot study, only simple and feasible preventive measures were applied. Therefore, the effectiveness of these measures was therefore not high and controversial.

Another issue that needs further discussion here is about the educational interventions that were heavily used in this study. The field of occupational health in general and work-related MSDs has received little attention in Vietnam. That is, the first thing we did is in order to raise the awareness of nurses about this content. From that, they can understand the nature and importance of work-related MSDs, and how do they negatively affect the health of workers. Once they had the knowledge, we conducted training on daily work practices to change behavior and protect themselves from the risk factors for MSDs. Besides, the suggested exercises to help strengthen the musculoskeletal system of nurses also contribute to reducing the risk of MSDs.

One of the limitations of the educational intervention in this study is that it only provides of theoretical information and knowledge to nurses but does not monitor the application of these measurements by nurses in their actual work. To ensure that we could provide the most complete knowledge of MSDs to nurses, as described in the research methods section, we flexibly used the recommendations of the World Health Organization (WHO) on the prevention of MSDs on the workplace [17] and on the prevention of MSDs on the hospital sector of the General Directorate of Labor Humanization in Belgium [18] and the Guideline for ergonomics for the prevention of MSDs of the Occupational Safety and Health Administration, US Department of Labor [19]. These recommendations cover most of the essential knowledge about MSDs and apply in the hospital setting to limit exposure to risk factors that can cause MSDs. In addition, repetition of these educational interventions is necessary to improve nurses' attitudes after different time periods [30, 32]. In fact, we repeated these interventions 3 times within the first 6 months. Educational interventions are not merely theoretical training to improve awareness and understanding but can also take other forms such as practical training in patient and manipulating with medical instruments [31] or training based on role-playing situations [30]. A study by Bos et al. [45] comparing approaches to knowledge education about MSDs for nurses, or as in Engels et al.'s study [46], showed that combining theoretical education with ergonomics will be more effective in the goal of reducing MSDs. We have tried to provide the most complete knowledge about MSDs for nurses. However, due to resource constraints (time, human resources, budget), it is also possible to negatively impact the effectiveness of these interventions. In addition, the application to practice in the daily work of Vietnamese nurses depends on many factors such as the workload, the will of each nurse, or the respect of the applied method. Furthermore, during the one-year intervention, hospitals have developed many innovative policies throughout the system based on the national patient satisfaction policy. This may have affected the interventional results.

Despite these limitations, it is undeniable that on the one hand, these first results open the premise for future intervention studies to prevent MSDs to be carried out with more effective methods; on the other hand, it will pave the way for new and appropriate policies to more effectively apply the interventions for nurses in particular and workers in other professions in general.

## 5. Conclusion

This study is the first to examine the effectiveness of several simple MSD prophylaxes in Vietnamese nurses. The effectiveness occurred probably on the prevalence of MSDs in the last 7 days at 4 anatomical sites (neck, shoulder/upper arm, wrists/hand, and lower back) between the 2 groups before and after the intervention. More in-depth studies are needed with a combination of measures over a longer period of time for more robust evidence on interventions.

## Figures and Tables

**Table 1:** Distribution of nurses by district hospital.

N°	Hospital	Hospital type	*N*
1	An Duong	Rural district	112
2	An Lao	Rural district	125
3	Cat Ba	Rural district	37
4	Cat Hai	Rural district	22
5	Duong Kinh	Urban district	45
6	Do Son	Urban district	33
7	Hai An	Urban district	30
8	Hong Bang	Urban district	87
9	Kien An	Urban district	45
10	Kien Thuy	Rural district	83
11	Le Chan	Urban district	85
12	Ngo Quyen	Urban district	130
13	Tien Lang	Rural district	85
14	Thuy Nguyen	Rural district	230
15	Vinh Bao	Rural district	130
Total			1 279

**Table 2:** Profiles of participants at baseline for the intervention and control group.

Characteristics	Intervention group, *n* = 162	Control group, *n* = 128	*p*
Age (year) (M ± SD)	33.6 ± 6.8	34.4 ± 6.6	0.321^∗^
Women (*n*, %)	144 (88.9)	108 (84.4)	0.258^∗∗^
BMI (*n*, %)			
< 18.5	9 (5.6)	12 (9.4)	0.459^∗∗^
18.5-24.9	144 (88.9)	109 (85.2)	
≥ 25	9 (5.6)	7 (5.5)	
Seniority			
Less than 5 years	44 (27.2)	17 (13.3)	0.054^∗∗^
From 5 to 10 years	43 (26.5)	45 (35.2)	
From 10 to 15 years	40 (24.7)	40 (31.3)	
More than 15 years	35 (21.6)	26 (20.3)	
Have a history of musculoskeletal diseases (*n*, %)	38 (23.5)	22 (17.2)	0.191^∗∗^

^∗^Student's *t*-test with two independent samples. ^∗∗^Chi2 test with two independent samples.

**Table 3:** Difference in the prevalence of MSDs (at least one of the 9 locations) between the two groups at two points in time (before and after intervention).

Independent variables		In the last 12 months			In the last 7 days		*p*
		*β*-Exponential (OR)	95% confidence interval	*p*	*β*-Exponential (OR)	95% confidence interval	
Group	Intervention	1	—	—	1	—	—
	Control	1.6	0.9-2.6	0.059	1.9	1.1–3.3	0.016
Intervention	Before	1	—	—	1	—	—
	After	1.9	1.3-2.9	0.001	2.0	1.3–3.0	0.002
Gender (women)		1.1	0.6-2.1	0.776	0.6	0.3-1.1	0.118
Age		1.1	0.9-1.1	0.003	1.0	0.9-1.1	0.106
BMI		1.0	0.9-1.1	0.721	1.0	0.9-1.1	0.927
History of musculoskeletal diseases (yes)		2.1	1.3-3.3	0.001	2.7	1.7-4.4	<0.001

Binary dependent variables: MSDs in the last 12 months (yes/no) and in the last 7 days (yes/no). Method used: GEE: generalized estimating equation.

**Table 4:** Difference in the prevalence of MSDs at each anatomical site between the two groups before and after intervention.

Independent variables		Neck			Shoulder/upper arm			Wrist/hand			Lower back		
		OR	95% CI	*p*	OR	95% CI	*p*	OR	95% CI	*p*	OR	95% CI	*p*
Group	Intervention	1	—	—	1	—	—	1	—	—	1	—	—
	Control	1.9	1.1-3.3	0.013	2.5	1.2–4.9	0.011	2.5	1.1–6.1	0.038	2.0	1.2–3.3	0.009
Intervention	Before	1	—	—	1	—	—	1	—	—	1	—	—
	After	2.1	1.3-3.5	0.002	2.3	1.2–4.5	0.015	2.4	1.1–5.2	0.024	1.3	0.9–1.9	0.216
Gender (women)		1.8	0.9-3.6	0.076	1.0	0.5–2.2	0.915	1.3	0.5–3.4	0.664	1.0	0.5–2.0	0.969
Age		1.04	1.0-1.1	0.019	1.1	1.0–1.1	0.001	1.0	1.0–1.1	0.053	1.0	1.0–1.1	0.089
BMI		1.0	0.9-1.1	0.533	1.0	0.9–1.1	0.719	1.1	0.9–1.3	0.345	1.0	0.9–1.1	0.418
History of musculoskeletal diseases (yes)		1.9	1.2-2.9	0.007	1.5	0.9–2.6	0.121	1.4	0.7–2.8	0.301	2.3	1.4–3.8	0.002

Binary dependent variables: MSDs in the neck, shoulder/upper arm, wrist/hand, and lower back in the last 7 days (yes/no). Method used: GEE: generalized estimating equation. For the remaining anatomical sites: *p* = 0.590 for the elbow/forearm, *p* = 0.328 for the upper back, *p* = 0.434 for the hip/thigh, *p* = 0.195 for the knee/lower leg, and *p* = 0.658 for the ankle/feet.

**Table 5:** Difference in quality of life and psychological distress scores between the two groups before and after intervention.

Independent variables		In the last 12 months			In the last 7 days		
		*β*	95% confidence interval	*p*	*β*	95% confidence interval	*p*
Group	Intervention	1	—	—	1	—	—
	Control	-0.8	-2.4; 0.8	0.344	0.1	-0.6; 0.8	0.789
Intervention	Before	1	—	—	1	—	—
	After	-2.6	-3.8; -1.4	<0.001	0.7	0.1; 1.3	0.019
Gender (women)		-1.4	-3.6; 0.8	0.222	-0.7	-1.6; 0.3	0.172
Age		0.003	-0.1; 0.1	0.956	-0.05	-0.1; 1.0	0.047
BMI		0.2	-0.1; 0.5	0.161	-0.1	-0.3; 0.1	0.221
History of musculoskeletal diseases (yes)		-2.5	-4.3; -0.7	0.007	1.1	0.3; 1.9	0.007

Continuous independent variable: quality of life score and psychological distress score. Method used: GEE: generalized estimating equation.

## Data Availability

The SPSS data used to support the findings of this study are available upon request from the corresponding author.
